# Multiobjective Optimization of Heat-Treated Copper Tool Electrode on EMM Process Using Artificial Bee Colony (ABC) Algorithm

**DOI:** 10.3390/ma15144831

**Published:** 2022-07-11

**Authors:** Geethapriyan Thangamani, Muthuramalingam Thangaraj, Khaja Moiduddin, Hisham Alkhalefah, Sivakumar Mahalingam, Panagiotis Karmiris-Obratański

**Affiliations:** 1 Department of Mechanical Engineering, Indian Institute of Technology Indore, Indore 453552, India; devimani.priyan18@gmail.com; 2 Department of Mechanical Engineering, SRM Institute of Science and Technology, Chennai 603203, India; 3 Department of Mechatronics Engineering, SRM Institute of Science and Technology, Chennai 603203, India; 4 Department of Industrial Engineering, College of Engineering, King Saud University, P.O. Box 800, Riyadh 11421, Saudi Arabia; halkhalefah@ksu.edu.sa; 5 Department of Mechanical Engineering, Vel Tech Rangarajan Sagunthala R&D Institute of Science and Technology, Chennai 600062, India; lawan.sisa@gmail.com; 6 Department of Manufacturing Systems, Faculty of Mechanical Engineering and Robotics, AGH University of Science and Technology, 30-059 Cracow, Poland; karmiris@agh.edu.pl; 7 Laboratory of Manufacturing Technology, School of Mechanical Engineering, National Technical University of Athens, 157 80 Athens, Greece

**Keywords:** EMM, surface, optimization, machining, ABC

## Abstract

Electrochemical micromachining (EMM) is a plausible method for manufacturing high accuracy and precision microscale components in a broad range of materials. EMM is commonly utilized to manufacture turbine blades for automobiles and aircrafts. In this present study, the EMM process was performed with a heat-treated copper tool electrode on aluminum 8011 alloy. The process parameters such as voltage, concentration of electrolyte, frequency, and duty factor were varied to analyze the effect of a heat-treated electrode on material removal rate (MRR), overcut, conicity, and circularity. It was observed that high MRR was obtained with lower overcut with an annealed electrode. The better conicity and circularity were obtained with a quenched electrode compared to other heat-treated and untreated tool electrodes. The artificial bee’s colony (ABC) algorithm was used to identify the optimum parameters and, finally, the confirmation test was carried out to evaluate the error difference on the machining process. The optimum combination of input process parameters found using TOPSIS and ABC algorithm for the EMM process are voltage (14 V), electrolyte concentration (30 g/L), frequency (60 Hz), and duty cycle (33%) for the annealed tool electrode and voltage (14 V), electrolyte concentration (20 g/L), frequency (70 Hz), and duty cycle (33%) for the quenched tool electrode. It was confirmed that 95% of accurate response values were proven under the optimum parameter combination.

## 1. Introduction

Due to increased demand for miniaturized and portable equipment, the need for precision parts has severely proliferated; and to fulfill these needs, various advanced machining techniques have been developed. One such technique is electrochemical machining (EMM), which leads to a great development and demand due to its versatile applications and benefits. It offers outstanding machinability, negligible tool wear, featureless surface finish, and economic efficiency. EMM has potential to machine any complicated shape on a workpiece which is capable of conducting electricity [1,2]. EMM is considered as a leading nonconventional machining technique as the machining rate is high and amount of precision is high [3]. EMM is very frequently used in aerospace, automotive, defense, electronic, and biomedical industries to produce miniaturized components [4]. The EMM process works on the principle of Michael Faraday’s laws of electrolysis. It involves the eroding of the anode by dissolution in the electrolyte. In the EMM process, the workpiece acts as the anode and the tool acts as the cathode. The dissolution of the workpiece starts when voltage is applied on the electrolytic cell by the movement of negative ions to the anode and positive ions to the cathode. The material which is removed from the workpiece forms a precipitate in the electrolyte [5]. A lot of research [6,7,8] was performed to analyze the effect of electrolytes while very little has been carried out to study the behavior of the tool and its effects on the EMM process. The interelectrode gap (IEG) equilibrium is sustained using sensing and control of potential difference across electrode and specimen [9,10]. Carbon fiber could be used as tool electrodes in the EMM process. It was found that a tool made up of PAN-based fiber is most suitable for the EMM process as it produces minimum defects [11]. A dual pole tool was employed to enhance the accuracy due to the localization of the electric field in between the electrode gap [12]. The effect of the shape of the tooltip was studied on the EMM process and concluded that a truncated tip increases the rate of machining by a factor of 4.4 while a rounded tip decreases the overcut by a factor of 1.7 when compared against a flat-tipped tool [13]. The coated tool has improved the performance of both the traditional and nontraditional machining process. It was observed that higher material erosion is possible with a nickel-coated copper electrode, whereas lower surface roughness is possible with a chromium-coated electrode [14,15,16]. The influence of the size of the tool was examined during the EMM process and a reduction in machining rate with an increase in the size of tool was reported [17]. It was concluded that the semicylindrical tool with ultrasonic vibrations offered better precision and rate of machining when compared to the conventional tool cathode [18]. The effects of copper and brass tools were examined in the EMM process and it was deduced that the copper tool produced 20.91% more MRR and 29.65% more radial overcut than the brass tool [19]. The process mechanism of electrochemical anodic dissolution can be modified by many approaches such as changing pulse pulses, the utilization of unconventional tools, and controlling of the processes. In the present study, an endeavor was made to utilize the optimization approaches in the electrochemical machining process. Since the process contains more than one response parameter, multiresponse decision making (MCDM) needed to be implemented [20]. To examine the data for interpretation using the RSM technique, a solid knowledge is required [21]. The assignment of weights is a simple procedure that is very simple to use. Its forecast accuracy is, however, not very good. According to the capacity of the process control variables, TGRA requires the lengthy and difficult procedure of selecting the grey coefficient [22]. Better performance prediction accuracy is offered by the ANN technique. However, it necessitates intricate computations and the use of optimization techniques, both of which have a significant computational cost [23]. Taguchi-data-envelopment-analysis-based-ranking (DEAR) could not applied for a study which contains only lower than better response parameters [24,25]. The proposed problem is involved with multiparameters and multiresponses in different scale levels and contradictory objectives which turns the problem into nonpolynomial hard nature. Hence, we introduced one of the metaheuristic algorithms, the ‘ABC algorithm’, in the present study due to its ease of use and high degree of accuracy. The present study focused on the effect of heat-treated tool electrodes on machining aluminum 8011 and the process parameters were optimized using the artificial bee’s colony algorithm.

The detailed survey illustrated that only few multiresponse decision-making (MCDM) were available with the machining of aluminum alloy using the EMM process. It was also found that little attention was given to optimizing the surface quality performance measures related to the EMM process on machining such an alloy. Hence, the present investigation was performed. In the present attempt, the artificial bee colony (ABC) methodology was performed for enhancing the surface performance measures on drilling aluminum 8011 alloy in the EMM process. The important aims of the investigation on machinability using different process factors were as follows:

1. To calculate the optimal factors for obtaining better multiple surface quality measures using the ABC technique.
2. To assess the influence of input factors on surface measures.
3. To inspect the surface quality at optimal levels in the process.

## 2. Materials and Methods 

The EMM setup comprises the electrical power unit, the machining chamber, the computer control system, and the electrolyte pumping system. The machining chamber contains the workpiece (anode) and tool (cathode). The workpiece is fixed using a holder in a machining chamber which is airtight and resistant to corrosion. It is equipped with a window to monitor the machining. The tool is made to move near the job with the help of a control panel equipped with press buttons and table lifting arrangement which helps in the interelectrode gap maintenance. A microcontroller governs the progress and maneuvers the tool vertically with the help of a servo motor. A control unit was used to vary the process parameters such as voltage, current, and feed rate. The selection of process factors is described in Table 1. Due to the electrochemical and chemical reactions occurring between anode and cathode, the removal of metal takes place in the form of sludge [26,27,28]. The workpiece used in this study was aluminum 8011, since it has high corrosion resistance, heat resistance, and high loading capacity [29,30,31]. The tool used for this study was copper due to its properties such as chemical inertness, good conductivity, and resistance to corrosion. The heat treatment involved performing annealing, normalizing, and quenching on the tool. Heat treatment processes were carried out at a temperature of 400 °C for the soaking time of 60 min. An aqueous solution of NaNO_3_ was chosen as an electrolyte due to its lower throwing power, high metal removal rate (MRR), and passivity of the alloy [32,33,34].

MRR is expressed as metal removed per unit time and can be denoted by g/min.

(1) $$\mathrm{MRR}=\frac{\mathrm{Difference}\;\mathrm{in}\;\mathrm{the}\;\mathrm{weight}\;\mathrm{of}\;\mathrm{workpiece}\;\mathrm{pre}\;\mathrm{and}\;\mathrm{post}\;\mathrm{EMM}\;\mathrm{process}}{\mathrm{Machining}\;\mathrm{Time}}$$

Overcut is the space between the tool and the machined hole measured in micrometers.

(2) $$\mathrm{Overcut}=\frac{\mathrm{Diameter}\;\mathrm{of}\;\mathrm{the}\;\mathrm{hole}\;\mathrm{at}\;\mathrm{entry}\;-\;\mathrm{Tool}\;\mathrm{Diameter}}{2}$$

Conicity is the variation between a real and ideal cylindrical surface. It is a blanket tolerance that results in a feature lying between concentric cylinders or coaxial cylinders. It is expressed in percentage as follows:

(3) $$\mathrm{Conicity}=\frac{\mathrm{Diameter}\;\mathrm{at}\;\mathrm{entry}-\mathrm{Diameter}\;\mathrm{at}\;\mathrm{exit}}{2\times \;\mathrm{thickness}\;\mathrm{of}\;\mathrm{workpiece}}$$

Circularity is the cross-sectional evaluation of the feature to determine if the circular surface lies between the tolerance zone indicated by two concentric circles, which is measured in micrometers.

(4) Circularity= Maximum Diameter − Minimum Diameter

### ABC Algorithm

In this present work, the influence of voltage (*V*), electrolyte concentration (*EC*), frequency (*F*), and duty cycle (*DC*) on different response values such as the material removal rate (*MRR*), overcut (*OC*), conicity (*CC*), and circularity (*CL*) were studied for an annealed and a quenched tool electrode using Taguchi’s L_9_ orthogonal array. Apart from that, multiple linear regression equations were established for the above responses and the optimum parameters and their corresponding response values have been obtained by implementing the ABC algorithm. Since four different responses/objective values were involved in this work, it was treated as a multiobjective optimization problem. Moreover, the objectives were of both a maximization and a minimization nature and of different magnitudes. The TOPSIS method was proposed in this work to convert multiobjectives into a single objective value. This converted single objective value was considered as the fitness function for the ABC algorithm. The lower and upper limits mentioned below for each parameter were considered as constraints in this algorithm.

(5) 10≤V≤1420≤EC≤3050≤F≤7033≤DC≤66

Table 2 illustrates different levels of parameter values based on which of the experiments were conducted and the response values were measured for the annealed tool electrode.

(6) $$rv_{j}=C_{0}+C_{1}V+C_{2}EC+C_{3}F+C_{4}\mathrm{DC}$$

Equation (6) represents a linear regression equation (LRE) used in this work which was constructed for the data provided in the above Table 2 using Minitab software. The coefficients of regression equations for the different response values are listed in Table 3. The experimental details and the coefficients of LRE for the quenched tool electrode are presented in Table 4 and Table 5, respectively. The lower and upper limits of each parameter are represented in Table 6.

**Figure 1:**
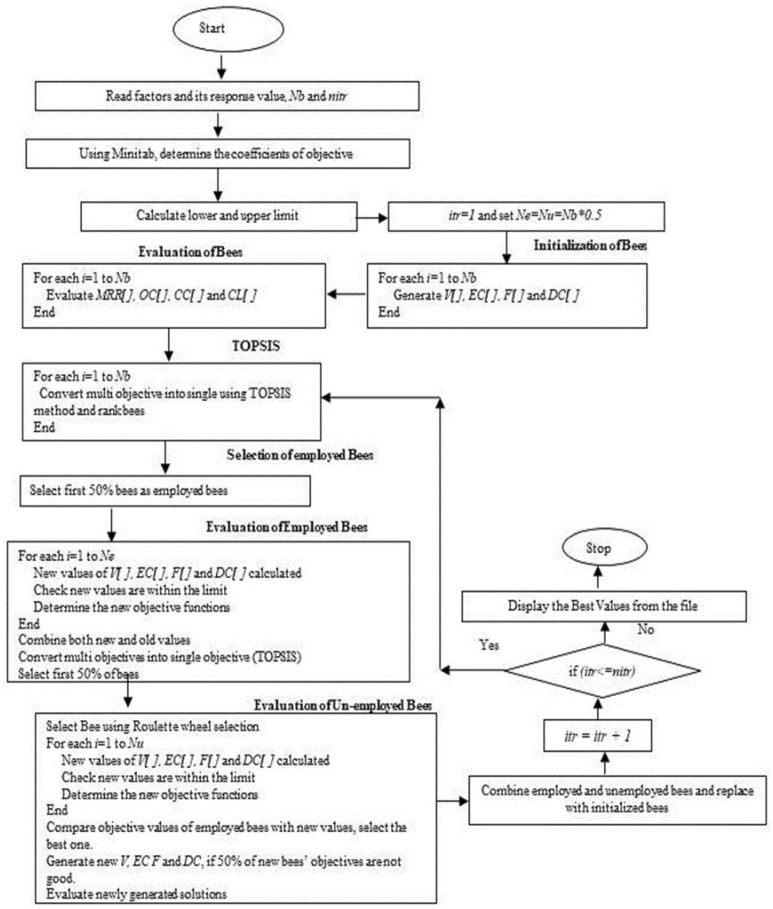
Implementation of ABC algorithm.

The implementation flow diagram for obtaining the optimum response values is presented in Figure 1, and the step-by-step procedure is demonstrated [35,36] in Appendix A. Table 7 represents the list of ABC parameters and their value considered in this work.

## 3. Results and Discussion

### 3.1. Influence of Heat Treatment on Copper Tool Electrode

**Figure 2:**
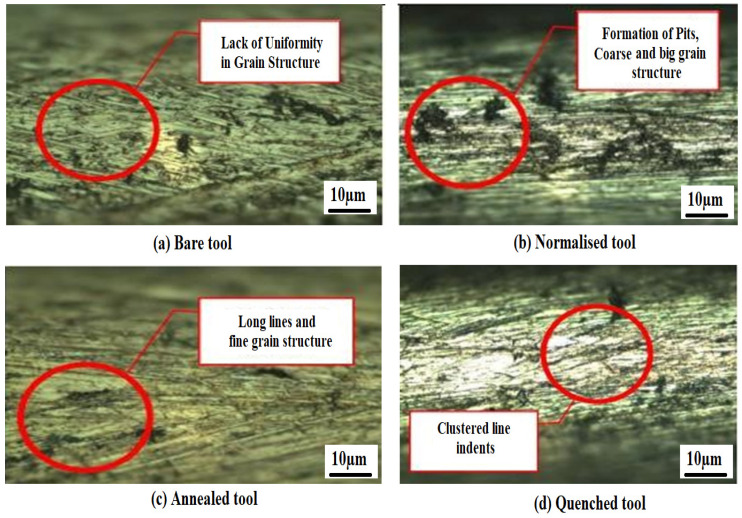
Steps’ effect of heat treatment on tool electrode. (**a**) Bare tool; (**b**) Normalized tool; (**c**) Annealed tool; (**d**) Quenched tool.

**Figure 3:**
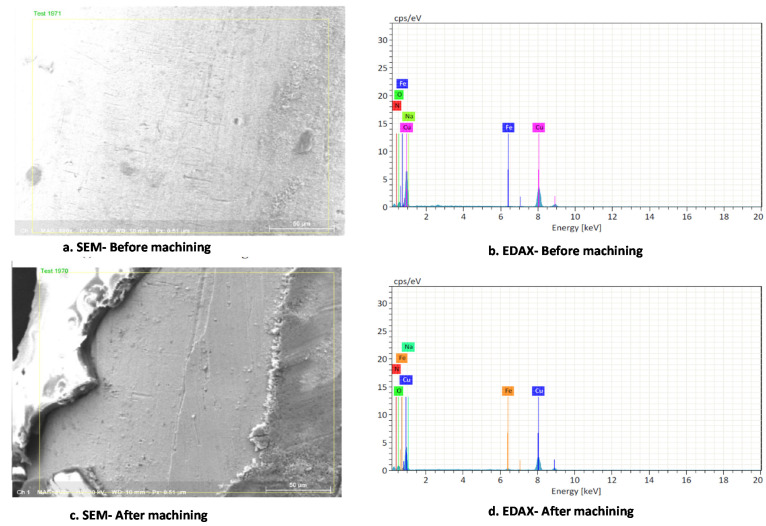
SEM and EDAX analysis of tool electrode before and after drilling process. (**a**) SEM-Before machining; (**b**) EDAX-Before machining; (**c**) SEM-After machining; (**d**) EDAX-After machining.

Figure 2 shows the microstructure of the bare and heat-treated copper tool electrode used for the electrochemical micromachining process. A lack of uniformity in grain structure was observed in the bare copper tool electrode. The occurrence of pits was seen after normalizing because pitting resistance reduced due to the exposure of the material to the atmosphere during cooling. Formation of long lines was observed in the annealed specimen along with some uniformity [37,38]. Smaller grain size was also observed in the annealed specimen due to the slow rate of cooling which leads to better compression in the material. Line indents were observed on the quenched specimen. Clustered line formation was visible on the surface of the specimen as a consequence of rapid cooling due to which solute atoms that precipitate on the grain force the vacancies to migrate into disordered regions. The loss of vacancies on the structure leads to a clustered grain structure [39,40]. Figure 3 shows the scanning electron microscope (SEM) and energy dispersive X-ray (EDAX) analysis of the tool electrode before and after the drilling process. The EDAX analysis was performed under area mode. It was observed that the percentage of copper tool electrode could be reduced owing to the presence of newer substances released from the workpiece specimens.

### 3.2. Influence of Process Parameters and Heat-Treated Tool Electrode on MRR

**Figure 4:**
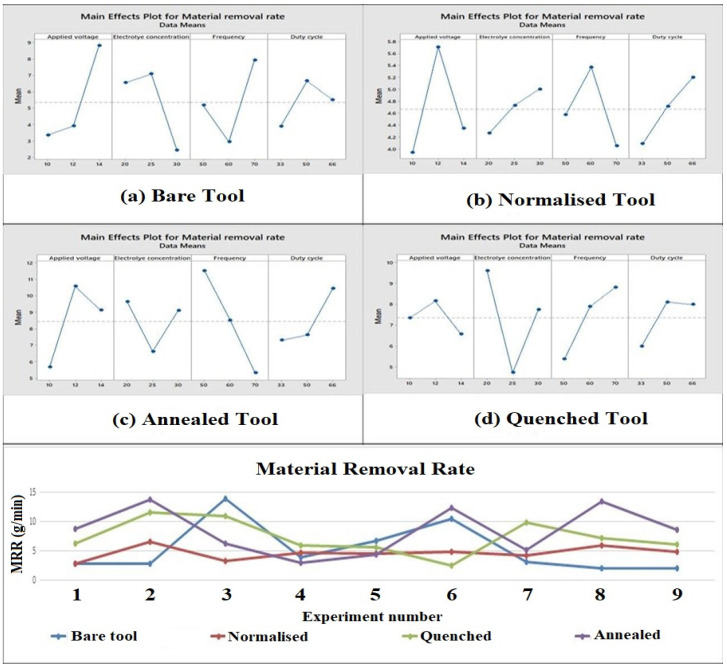
Main effect plots and comparison plot of MRR for tool electrodes. (**a**) Bare tool; (**b**) Normalized tool; (**c**) Annealed tool; (**d**) Quenched tool.

From Figure 4 (main effect plot), the maximum deviation was observed in the bare and normalized copper tool electrode was applied voltage. It is the most influential parameter in these cases. MRR increased as applied voltage increased. In the case of the annealed tool electrode, the frequency showed the most deviation in the machining process. The frequency depends upon the duration of the pulse on and pulse off time. Therefore, current will be supplied for a longer period of time leading to high MRR as machining occurs only when current is being supplied. This statement is further supported by the main effect plot of the annealed tool as the MRR could be decreased with higher frequency.

In the case of the quenched tool electrode, electrolyte concentration showed the most deviation, thus suggesting that it was the most influential parameter because a higher electrolyte concentration leads to the generation of a larger number of ions during electrochemical machining. This leads to a larger ionization which causes high erosion and conductivity for creating larger material removal [41,42]. It was seen that the annealed tool has the highest MRR because the annealed tool has the smallest grain structure compared to other electrodes which facilitates a faster dissolution and removal of particles from the workpiece, resulting in the increase in MRR. The bare tool was noticed to have a lesser MRR because the bare tool has a large grain size among all the electrodes due to which the dissolution and eroding of material from the work specimen takes a longer time. It was found that a 57% higher material removal was obtained with the annealed tool than the bare tool.

### 3.3. Influence of Process Parameters and Heat-Treated Tool Electrode on Overcut

**Figure 5:**
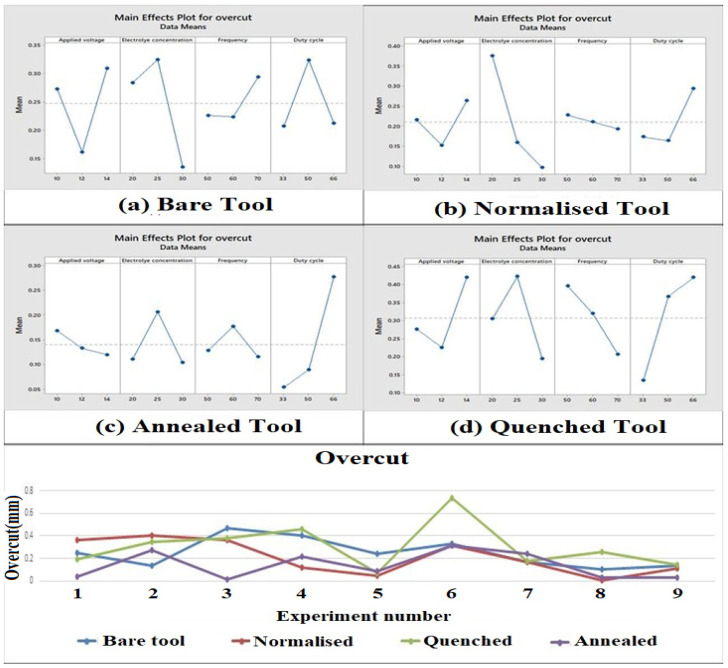
Main effect plots and comparison plot of overcut for tool electrodes. (**a**) Bare tool; (**b**) Normalized tool; (**c**) Annealed tool; (**d**) Quenched tool.

It is apparent from Figure 5 that electrolyte concentration shows the most deviation in the main effect plots of bare and normalized tool electrode. The quantity of NO^3−^ in the solution was increased due to the higher concentration of electrolyte which increases the localization of ions resulting in less overcut. Duty cycle shows the most deviation for the annealed and quenched tool electrodes. It can be seen from the main effect plot that the overcut was high when the duty cycle was high because, as the pulse on time increases, the current flow between the electrode and workpiece increases. A higher current flow leads to a higher dissolution of ions. This limits the magnitude of the current, thereby decreasing the dissolution rate. This leads to achieving a higher overcut owing to a higher side current and lower localization effect [43,44]. It is evident that the annealed tool had a lesser overcut which is desirable in EMM as low overcut indicates higher precision in machining. This is because the annealed tool has a fine grain structure due to furnace cooling which improves the surface finish of the tool electrode. The quenched tool has a nonuniform grain structure which leads to a higher erosion rate during machining. The overcut of the annealed tool electrode was 26.05% less than the bare tool.

### 3.4. Influence of Process Parameters and Heat-Treated Tool Electrode on Conicity 

**Figure 6:**
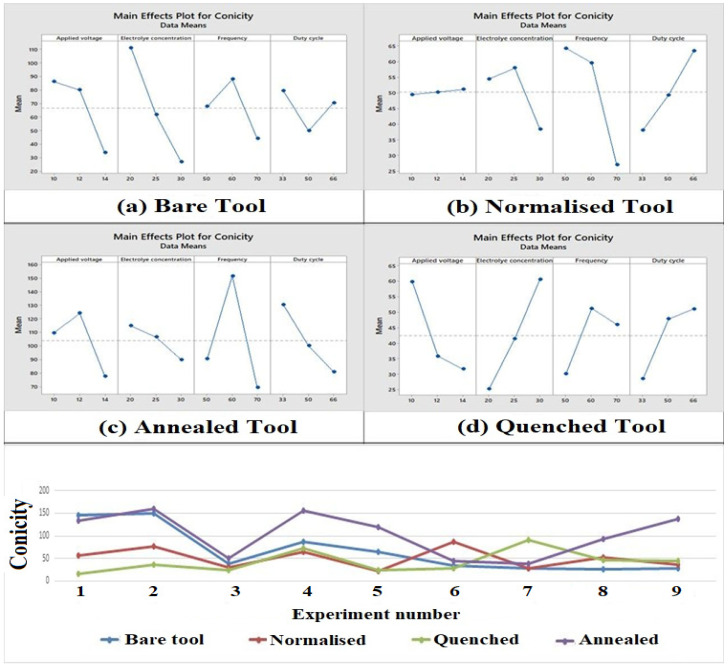
Main effect plots and comparison plot of conicity for tool electrodes. (**a**) Bare tool; (**b**) Normalized tool; (**c**) Annealed tool; (**d**) Quenched tool.

The electrolyte concentration was observed to be the most influential parameter for the bare and quenched tool electrodes as shown in Figure 6. When the electrolyte concentration is less, the ionization rate reduces due to the large grain size of the material which in turn leads to an increase in conicity. For the normalized and annealed tools, the frequency was observed to be the most influential parameter. It is evident from the main effect plot that conicity increases with an increase in frequency which implies a decrease in pulse duration. It is noted that the duty cycle ratio is also high when the frequency is high. This shows that the larger pulse could, in time, lead to a higher conicity [45]. It can be seen that the quenched tool had a better conicity due to rapid cooling which leads to a more uniform structure, thus improving its conicity of the machined through hole. The quenched tool electrode had 36.38% better conicity than the untreated tool electrode.

### 3.5. Influence of Process Parameters and Heat-Treated Tool Electrode on Circularity

**Figure 7:**
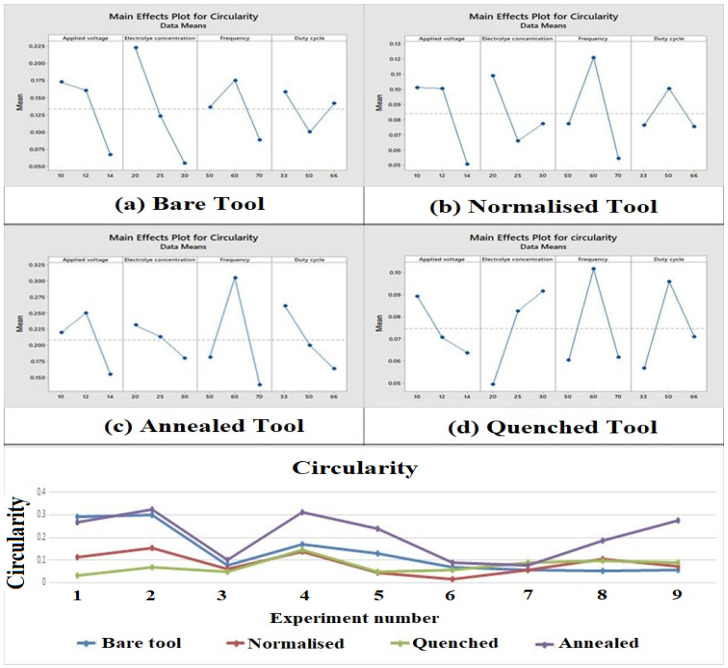
Main effect plots and comparison plot of circularity for tool electrodes. (**a**) Bare tool; (**b**) Normalized tool; (**c**) Annealed tool; (**d**) Quenched tool.

It is observed from Figure 7, the most influential parameter was electrolyte concentration for the bare and quenched tool electrode. When the electrolyte concentration is lower, then the ionization rate reduces due to the large grain size of the material, which in turn leads to an increase in the circularity of the hole machined. For the normalized and annealed tool, the frequency was inferred to be the most influential parameter. It can be seen from the main effect plot that with the raise in frequency, the circularity increased because an increment in frequency implies a decrease in pulse duration and an increase in the ionization which improves the circularity [46]. It is evident that the normalized tool and the quenched tool electrode had a low circularity deviation. This is because the quenched and normalized tools were rapidly cooled which leads to a more uniform structure, thus bettering its circularity of the machined through hole. The quenched tool electrode had 44.13% lesser circularity than the untreated tool electrode.

## 4. Analysis of Variance

Analysis of variance is a statistically based decision-making tool used to detect any deviations in the mean performance of a group of items that have been tested. ANOVA compares the mean square against the estimate of the experimental errors at set confidence levels, thus aiding in the identification of significance of the main factors and their interactions in a study [47,48].

(7) $$SS_{T}=\sum_{i=1}^{n}{(〖n_{i}〗-n_{m})}^{2}$$

where *n* is the no. of experiment and *n_i_* is the mean S/N ratio

### 4.1. ANOVA for MRR

From Table 8, the most influential parameter for MRR in EMM using an annealed tool electrode was the frequency as it contributes to 44.61% of the sum of squares value. With an increase in frequency, the current supplied per cycle increases which leads to better machining. The table has been computed for the MRR of the annealed tool as it has performed a better MRR out of all the tool electrodes. The annealed tool electrode that was computed had an MRR that was 57% better than the untreated tool [49].

### 4.2. ANOVA for Overcut

From Table 9, the most influential parameter for overcut was duty cycle. Duty cycle contributed 45.91% to the sum of squares value. Duty cycle also determines the ratio of pulse ON and pulse OFF times which determines the duration of the current supplied per cycle. With an increase in duty cycle ratio, the machining rate increases as the duration of current supplied per cycle increases. The annealed tool had an overcut which was 26.05% less than that of the bare tool.

### 4.3. ANOVA for Conicity

From Table 10, the most influential parameter for conicity was electrolyte concentration. This is because electrolyte concentration determines the rate of ionization in an EMM process. When the electrolyte concentration increases, the ionization rate increases due to the presence of free ions which aids in machining. The quenched tool provided a conicity 36.06% better than the bare tool electrode.

### 4.4. ANOVA for Circularity

From Table 11, the electrolyte concentration was the most influential parameter for circularity. This is because a higher electrolyte concentration generates a larger ionization rate which in turn leads to a high circularity. When electrolyte concentration increases, the number of free ions present in the solution increases and results in better machining and localization of the current. The quenched tool electrode had a circularity that was 44.13% better than bare tool electrode.

## 5. Optimum Process Parameters and Their Responses

**Figure 8:**
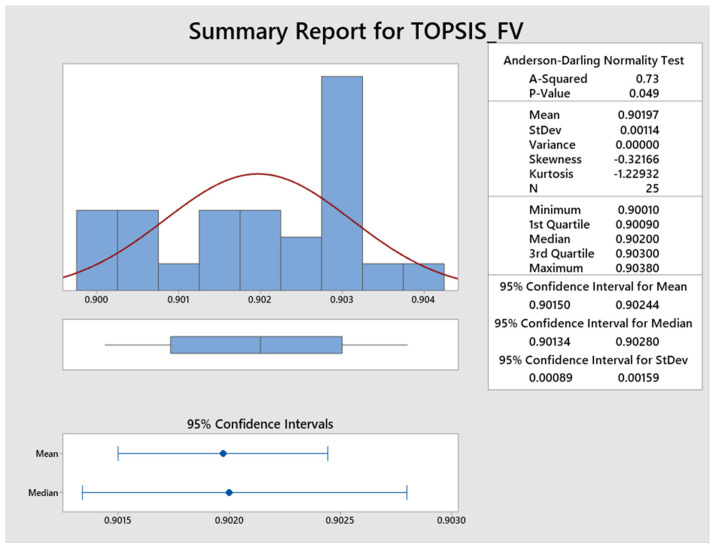
Statistical analysis of 25 runs.

**Figure 9:**
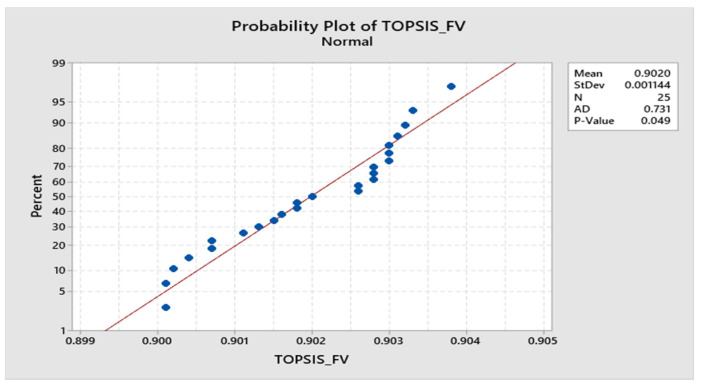
Normality test of 25 runs.

The program executed 25 runs and the statistical analysis of the results is shown in Figure 8. It is confirmed by the *p*-Value of 0.049 that the fitness values obtained by 25 runs were in the normal distribution range; hence, the optimum parameters obtained by ABC algorithms are acceptable. The normality test of 25 runs is represented in Figure 9 which also supports the acceptability of the ABC algorithm’s results and the model proposed is adequate to demonstrate the relationship between various inputs to response values.

**Figure 10:**
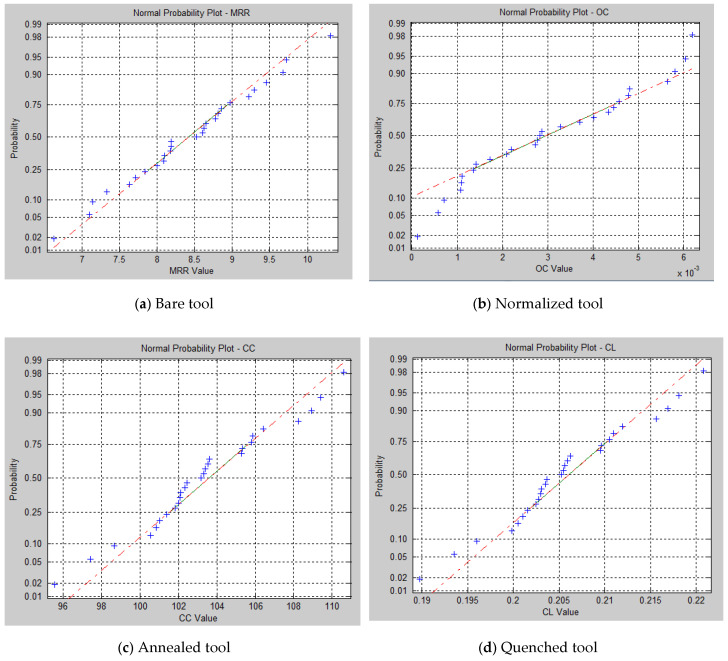
Normal probability plot for response values. (**a**) Bare tool; (**b**) Normalized tool; (**c**) Annealed tool; (**d**) Quenched tool.

**Figure 11:**
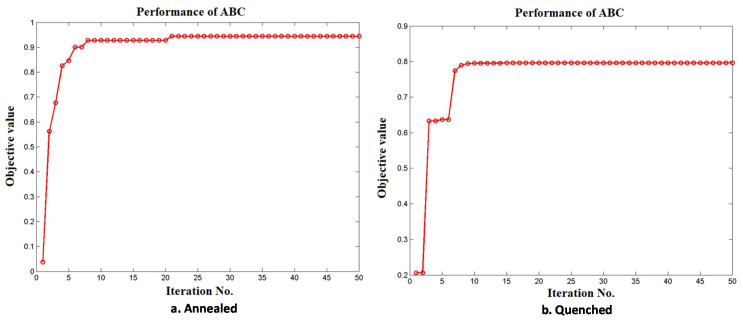
Convergence plot of ABC algorithm (**a**) Annealed; (**b**) Quenched.

Figure 10 represents the normal probability plot drawn for the output of response values obtained by implementing the ABC algorithm. Figure 11 shows the convergence plot of the ABC algorithm. It shows that quick convergence of the algorithm was obtained in both the annealed and the quenched tool electrode. The optimum values of *V*, *EC*, *F*, and *DC* for the maximum value of the MRR and the minimum values of *OC*, *CC*, and *CL* are shown in Table 12.

Table 13 represents the response values obtained through the confirmation test conducted for the optimum parameter setting provided in Table 12. It was confirmed that less than 5% variation in the response values is proved and the proposed MCDM models and the optimum parameter values are acceptable.

## 6. Conclusions

In this present study, aluminum 8011 was machined by the EMM process using various heat-treated copper tool electrodes and the process parameters were optimized by TOPSIS and ABC algorithm. The input characteristics such as voltage, concentration of electrolyte, frequency, and duty factor were suitably varied to analyse their effect on the response characteristics such as MRR, overcut, circularity, and conicity. From the experiment conducted and the results obtained, the following conclusions were drawn.

(i) The annealed tool electrode created a higher MRR than the untreated, normalized, and quenched tool electrodes because the annealed tool electrode has a fine grain structure due to the slower rate of cooling and easily dissolves in the electrolyte.
(ii) The annealed tool electrode generated better overcut than the untreated, normalized, and quenched tool electrodes because the annealed tool has a smaller grain structure due to furnace cooling which improves the surface finish of the tool electrode.
(iii) Electrolyte concentration was the most influential parameter for the bare tool as it determines the rate of ionization due to the presence of free ions in the electrolyte.
(iv) The optimum combination of input process parameters found using TOPSIS and the ABC algorithm for the EMM process are voltage (14 V), electrolyte concentration (30 g/L), frequency (60 Hz), and duty cycle (33%) for the annealed tool electrode and voltage (14 V), electrolyte concentration (20 g/L), frequency (70 Hz), and duty cycle (33%) for the quenched tool electrode.

## Figures and Tables

**Table 1:** Selection of process factors.

Process Parameters	Level I	Level II	Level III
Voltage (V)	10	12	14
Concentration of Electrolyte (g/L)	20	25	30
Frequency (Hz)	50	60	70
Duty Factor (%)	33	50	66

**Table 2:** Details of experiments (E)—parameters and responses—annealed.

E.No.	*V*	*EC*	*F*	*DC*	*MRR*	*OC*	*CC*	*CL*
1	10	20	50	33	8.767	0.0405	134.0	0.269
2	12	20	60	66	13.804	0.2770	160.0	0.325
3	14	20	70	50	6.354	0.0145	50.5	0.100
4	10	25	60	50	3.076	0.2205	156.5	0.313
5	12	25	70	33	4.444	0.0875	119.0	0.238
6	14	25	50	66	12.345	0.3105	44.5	0.089
7	10	30	70	66	5.181	0.2430	38.5	0.077
8	12	30	50	50	13.453	0.0335	93.5	0.187
9	14	30	60	33	8.695	0.0335	138.0	0.276

**Table 3:** Coefficients of linear regression equations—annealed.

*rv_j_*	Coefficients				
	*C*_0_	*C*_1_	*C*_2_	*C*_3_	*C*_4_
*MRR*	13.3151	0.8642	−0.0532	−0.3098	0.0944
*OC*	0.0102	−0.0121	−0.0007	−0.0007	0.0067
*CC*	400.31	−8	−2.4833	−1.0667	−1.4979
*CL*	0.8074	−0.0162	−0.0051	−0.0022	−0.003

**Table 4:** Details of experiments—parameters and responses—quenched.

E.No.	*V*	*EC*	*F*	*DC*	*MRR*	*OC*	*CC*	*CL*
1	10	20	50	33	6.231	0.1895	16.5	0.032
2	12	20	60	66	11.5700	0.3475	36.0	0.069
3	14	20	70	50	11.020	0.3765	23.5	0.047
4	10	25	60	50	6.000	0.4625	73.0	0.146
5	12	25	70	33	5.600	0.0675	24.5	0.048
6	14	25	50	66	2.612	0.7355	27.0	0.054
7	10	30	70	66	9.800	0.1745	90.0	0.090
8	12	30	50	50	7.294	0.2605	47.0	0.095
9	14	30	60	33	6.122	0.1465	44.5	0.090

**Table 5:** Coefficients of linear regression equations—quenched.

*rv_j_*	Coefficients				
	*C*_0_	*C*_1_	*C*_2_	*C*_3_	*C*_4_
MRR	0.9682	−0.1897	−0.1868	0.1714	0.0616
OC	0.2874	0.036	−0.0111	−0.0094	0.0087
CC	−42.5712	−7.0417	3.5167	0.7917	0.6866
CL	0.0192	−0.0064	0.0042	0.0001	0.0005

**Table 6:** Limits of parameters.

Limits	*V*	*EC*	*F*	*DC*
Lower	10	20	50	33
Upper	14	30	70	66

**Table 7:** Parameters of ABC algorithm.

Parameters	Value
No. of Bees (Population Bees)	30
No. of Employed Bees	15 (50% of total bees)
No. of Unemployed Bees	15 (50% of total bees)
Termination Criteria	50 iterations

**Table 8:** ANOVA of MRR for annealed tool.

Source	DF	Adj SS	Adj MS	F-Value	*p*-Value	Contribution
Regression	4	90.482	22.6205	2.35	0.214	
Applied voltage	1	17.923	17.9228	1.86	0.244	13.89
Electrolyte concentration	1	0.425	0.4245	0.04	0.844	0.32
Frequency	1	57.573	57.5732	5.97	0.071	44.61
Duty cycle	1	14.561	14.5614	1.51	0.286	11.28
Error	4	38.552	9.6380			
Total	8	129.034				

**Table 9:** ANOVA of overcut for annealed tool.

Source	DF	Adj SS	Adj MS	F-Value	*p*-Value	Contribution
Regression	4	0.077419	0.019355	2.01	0.258	
Applied voltage	1	0.003528	0.003528	0.37	0.578	3.04
Electrolyte concentration	1	0.000081	0.000081	0.01	0.932	0.06
Frequency	1	0.000260	0.000260	0.03	0.878	0.22
Duty cycle	1	0.073550	0.073550	7.62	0.051	45.91
Error	4	0.038601	0.009650			
Total	8	0.116020				

**Table 10:** ANOVA for conicity of quenched tool.

Source	DF	Adj SS	Adj MS	F-Value	*p*-Value	Contribution
Regression	4	4191.3	1047.8	6.32	0.051	
Applied voltage	1	1190.0	1190.0	7.18	0.055	24.56
Electrolyte concentration	1	1855.0	1855.0	11.19	0.029	38.28
Frequency	1	376.0	376.0	2.27	0.206	7.76
Duty cycle	1	770.2	770.2	4.65	0.097	15.89
Error	4	662.9	165.7			
Total	8	4854.2				

**Table 11:** ANOVA for circularity for quenched tool.

Source	DF	Adj SS	Adj MS	F-Value	*p*-Value	Contribution
Regression	4	0.004016	0.001004	0.70	0.629	
Applied voltage	1	0.000988	0.000988	0.69	0.452	10.156
Electrolyte concentration	1	0.002688	0.002688	1.88	0.242	27.63
Frequency	1	0.000003	0.000003	0.00	0.968	0.03
Duty cycle	1	0.000337	0.000337	0.24	0.653	3.46
Error	4	0.005713	0.001428			
Total	8	0.009728				

**Table 12:** Optimum parameter values for annealed and quenched process.

Type	*V*	*EC*	*F*	*DC*	*MRR*	*OC*	*CC*	*CL*
Annealed	13.9947	29.9974	59.0402	33.0503	8.64425	0.00136	101.379	0.20152
Quenched	13.4648	20.004	66.4348	33.0107	8.09388	0.20939	8.21947	0.03688

**Table 13:** Confirmation test for annealed and quenched process.

Type	*V*	*EC*	*F*	*DC*	*MRR*	*OC*	*CC*	*CL*
Annealed	14	30	60	33	8.82265	0.00122	96.582	0.18526
Quenched	14	20	70	33	8.42349	0.22548	7.8254	0.05125

## Data Availability

The data presented in this study are available from the corresponding author on reasonable request.

## Nomenclature

*rv_j_*	*j*th Response value
*j*	Index for response value
***C*_0_, *C*_1_, *C*_2_, *C*_3_** and ***C*_4_**	Coefficients of regression equation
*SS_T_*	Sum square total
*O_ij_*	ith bee’s *j*th response value
*i*	Index for bee
*N_ij_*	Normalized value of *i*th bee’s *j*th response
*A_ij_*	Performance value of *i*th bee’s *j*th response
*W_j_*	Weight of *j*th response
*P_j_*	Positive ideal solution of *j*th response
*M_j_*	Negative ideal solution of *j*th response
*SP_i_*	Positive ideal separation value of *i*th bee
*SM_i_*	Negative ideal separation value of *i*th bee
*OV_i_*	Relative closeness value of *i*th bee
*B.No.*	Bee number
*EBNo.*	Employed bee number
*UBNo.*	Unemployed bee number
*nb*	Number of bees
*Pr_i_*	Probability of *i*th bee
*CPr_i_*	Cumulative probability of *i*th bee

## Appendix A

### Appendix A.1. Step 1: Initialization

First, a set of random values between the lower and upper limits of each parameter are generated and assumed as initial artificial bees. For the demonstrated purpose, 10 bees are considered and illustrated in Table A1. Using Equation (5) and the coefficients presented in Table 6, the response values such as *MRR*, *OC*, *CC*, and *CL* are calculated for the generated initial values provided in Table A2. It is demonstrated for the first bee and the calculated response values are provided below.

$$MRR_{1}=13.3151+0.8642\times 13.26-0.0532\times 21.58-0.3098\times 63.11+0.0944\;\times 56.30=9.389$$

$$OC_{1}=0.0102-0.0121\times 13.26-0.0007\times 21.58-0.0007\times 63.11+0.0067\times 56.30=0.1698$$

$$CC_{1}=400.31-8\times 13.26-2.4833\times 21.58-1.0667\times 63.11-1.4979\times 56.30=89.0072$$

$$CL_{1}=0.8074-0.0162\times 13.26-0.00051\times 21.58-0.0022\times 63.11-0.003\times 56.30=0.1791$$

**Table A1:** Initialization of artificial bees.

B.No.	*V*	*EC*	*F*	*DC*
1	13.26	21.58	63.11	56.30
2	13.62	29.71	50.71	34.05
3	10.51	29.57	66.98	42.14
4	13.65	24.85	68.68	34.52
5	12.53	28.00	63.57	36.21
6	10.39	21.42	65.15	60.17
7	11.11	24.22	64.86	55.93
8	12.19	29.16	57.84	43.46
9	13.83	27.92	63.11	64.36
10	13.86	29.59	53.42	34.14

Similarly, all other bees’ response values are determined and shown in Table A2.

**Table A2:** Response values of bees.

B.No.	Objectives Matrix–*O_ij_*			
	*MRR*	*OC*	*CC*	*CL*
1	9.389	0.1698	89.0072	0.1791
2	11.0122	0.0183	112.4572	0.2241
3	4.0514	0.0997	108.2449	0.216
4	5.7761	0.0128	104.392	0.2082
5	6.3774	0.0388	108.491	0.2163
6	6.6521	0.2293	104.3681	0.2104
7	6.8185	0.1902	98.2969	0.1975
8	8.4807	0.0945	103.5985	0.2069
9	10.3072	0.2122	56.6152	0.1135
10	10.3912	0.0143	107.8225	0.2147

### Appendix A.2. Step 2: Evaluation

In this present work, the four objective values shown in the above Table A2 are involved and each in different scales and different objective types such as maximizing *MRR* and minimizing *OC*, *CC*, and *CL*. Hence, in this work, the TOPSIS (Technique for Order Preference by Similarity to Ideal Solution) method has been adopted to convert the multiobjectives into a single objective [50]. The step-by-step procedure of the algorithm is provided below.

Read alternate and objectives matrix—*O_ij_* with weights (*W_j_*) and types of objectives (2—maximization and 1—minimization) where *i* represents bee numbers and *j* represents objective/response numbers.

Normalized value of *O_ij_* is computed using Equation (A1).

(A1) $$N_{ij}=\frac{O_{ij}}{\sqrt{\sum_{i=1}^{m}O_{ij}^{2}}}$$

Performance matrix (*A_ij_*) is calculated based on Equation (A2). Table A3 represents the normalized values of objectives and its performance matrix.

(A2) $$A_{ij}=N_{ij}*W_{j}$$

The positive ideal (*P_j_*) and negative ideal solution (*M_j_*) based on either the maximization (Equation (A3)) or minimization (Equation (A4)) objective are determined and presented in Table A4.

(A3) $$\begin{array}{l} P_{j}=\overset{m}{\underset{i=1}{\max}}(A_{ij})\\ M_{j}=\overset{m}{\underset{i=1}{\min}}(A_{ij}) \end{array}$$

(A4) $$\begin{array}{l} P_{j}=\overset{m}{\underset{i=1}{\min}}(A_{ij})\\ M_{j}=\overset{m}{\underset{i=1}{\max}}(A_{ij}) \end{array}$$

By using Equations (A5) and (A6), the positive ideal (*SP_i_*) and negative ideal separation (*SM_i_)* are determined and shown in Table A4.

(A5) $$SP_{i}=\sqrt{\sum_{j=1}^{n}{\left(A_{ij}-P_{j}\right)}^{2}}$$

(A6) $$SM_{i}=\sqrt{\sum_{j=1}^{n}{\left(A_{ij}-M_{j}\right)}^{2}}$$

The relative closeness (*OV_i_*) value is calculated based on Equation (A7) and listed in Table A5.

(A7) $$OV_{i}=\frac{SM_{i}}{SP_{i}+SM_{i}}$$

Rank bees based on *OV_i_* in descending order and select the best one and its parameter in a file. The relative closeness value (*OV_i_*) expressed in Equation (A7) is considered as the fitness value in the ABC algorithm.

**Table A3:** Normalized and performance matrix.

B.No.	Normalized Value (*N_ij_*)				Performance Matrix (*A_ij_*)			
	*MRR*	*OC*	*CC*	*CL*	*MRR*	*OC*	*CC*	*CL*
1	0.3609	0.3962	0.28	0.2817	0.0902	0.099	0.07	0.0704
2	0.4233	0.0427	0.3537	0.3525	0.1058	0.0107	0.0884	0.0881
3	0.1557	0.2326	0.3405	0.3398	0.0389	0.0582	0.0851	0.0849
4	0.222	0.0299	0.3284	0.3275	0.0555	0.0075	0.0821	0.0819
5	0.2451	0.0905	0.3413	0.3402	0.0613	0.0226	0.0853	0.0851
6	0.2557	0.535	0.3283	0.331	0.0639	0.1338	0.0821	0.0827
7	0.2621	0.4438	0.3092	0.3107	0.0655	0.1109	0.0773	0.0777
8	0.326	0.2205	0.3259	0.3255	0.0815	0.0551	0.0815	0.0814
9	0.3962	0.4951	0.1781	0.1785	0.0991	0.1238	0.0445	0.0446
10	0.3994	0.0334	0.3392	0.3377	0.0999	0.0083	0.0848	0.0844

**Table A4:** Positive and negative ideal solutions.

P/M	*MRR*	*OC*	*CC*	*CL*
*P_j_*	0.1058	0.0075	0.0445	0.0446
*M_j_*	0.0389	0.1338	0.0884	0.0881

**Table A5:** Ideal, negative ideal and relative closeness values.

B.No.	*SP_i_*	*SM_i_*	*OV_i_*
1	0.0997	0.067	0.4019
2	0.0619	0.1401	0.6936
3	0.1016	0.0757	0.4271
4	0.073	0.1277	0.6362
5	0.0742	0.1134	0.6044
6	0.1434	0.0263	0.1552
7	0.1204	0.0382	0.2409
8	0.0747	0.0899	0.5463
9	0.1165	0.0868	0.4269
10	0.0569	0.1395	0.7102

### Appendix A.3. Step 3: Selection of Employed Bees

The employed bees are selected based on the relative closeness values provided in Table A5. Out of 10 bees, the top 5 bees are considered as employed bees. Table A6 represents the employed bees’ objective values.

**Table A6:** Employed bees.

B.No.	*V*	*EC*	*F*	*DC*	*MRR*	*OC*	*CC*	*CL*	*OV*	*EBNo.*
10	13.86	29.59	53.42	34.14	10.3912	0.0143	107.8225	0.2147	0.7102	1′
2	13.62	29.71	50.71	34.05	11.0122	0.0183	112.4572	0.2241	0.6936	2′
4	13.65	24.85	68.68	34.52	5.7761	0.0128	104.392	0.2082	0.6362	3′
5	12.53	28.00	63.57	36.21	6.3774	0.0388	108.491	0.2163	0.6044	4′
8	12.19	29.16	57.84	43.46	8.4807	0.0945	103.5985	0.2069	0.5463	5′

### Appendix A.4. Step 4: Searching of New Food by Employed Bees

In this phase, the new values (*n_ij_*) are generated using the following Equation (A8) where the difference between the current (*o_ij_*) and selected bee (*o_kj_*) values are multiplied by a random value (*R_ij_*) between −1 and 1. Table A7 illustrates the selected bees and random number for generating the new solutions. By substituting the values provided in Table A7 in Equation (A8), the new solutions are generated for employed bees and represented in Table A8.

(A8) $$n_{ij}=o_{ij}+R_{ij}(o_{ij}-o_{kj})$$

**Table A7:** Random number and selected bee for new solution.

EBNo.(*i*)	*R_ij_*				
	*V*	*EC*	*F*	*DC*	REBNo.(*k*)
1′	−0.1225	−0.0205	−0.4479	−0.0033	4′
2′	−0.2369	−0.1088	0.3594	0.9195	5′
3′	0.531	0.2926	0.3102	−0.3192	1′
4′	0.5904	0.4187	−0.6748	0.1705	5′
5′	−0.6263	0.5094	−0.762	−0.5524	2′

**Table A8:** New food by employed bees.

NEBNo.	New Parameter Value by Employed Bees				New Objective Values			
	*nV*	*nEC*	*nF*	*nDC*	*nMRR*	*nOC*	*nCC*	*nCL*
n1	13.70	29.56	57.97	34.14	10.3912	0.0143	107.8225	0.2147
n2	13.28	29.65	60.94	37.57	11.0122	0.0183	112.4572	0.2241
n3	13.54	23.47	55.15	34.40	5.7761	0.0128	104.392	0.2082
n4	12.73	27.52	59.71	34.97	6.3774	0.0388	108.491	0.2163
n5	13.09	28.88	52.41	38.26	8.4807	0.0945	103.5985	0.2069

Data from both Table A6 and Table A8 are combined together and then a new single objective is calculated using TOPSIS for each bee and sorted from maximum to minimum based on the single objective (Table A9) and the top 50% of bees are selected for next process. This is shown in Table A10 and considered as the outcome of the employed bees.

**Table A9:** New single objective values.

B.No.	*V*	*EC*	*F*	*DC*	*MRR*	*OC*	*CC*	*CL*	*OV*
1′	13.86	29.59	53.42	34.14	10.3912	0.0143	107.8225	0.2147	0.9362
2′	13.62	29.71	50.71	34.05	11.0122	0.0183	112.4572	0.2241	0.899
3′	13.65	24.85	68.68	34.52	5.7761	0.0128	104.392	0.2082	0.7696
4′	12.53	28.00	63.57	36.21	6.3774	0.0388	108.491	0.2163	0.6226
5′	12.19	29.16	57.84	43.46	8.4807	0.0945	103.5985	0.2069	0.1669
n1	13.70	29.56	57.97	34.14	8.8443	0.0133	104.3467	0.2077	0.8875
n2	13.28	29.65	60.94	37.57	7.8853	0.0393	99.1354	0.1973	0.6552
n3	13.54	23.47	55.15	34.40	9.9347	0.0232	123.3294	0.2468	0.8084
n4	12.73	27.52	59.71	34.97	7.6584	0.0309	114.0552	0.2276	0.7182
n5	13.09	28.88	52.41	38.26	10.4647	0.0525	110.6841	0.2209	0.543

**Table A10:** Outcome of employed bees.

B.No.	*V*	*EC*	*F*	*DC*	*MRR*	*OC*	*CC*	*CL*	*OV_i_*	*OEBNo.*
1′	13.86	29.59	53.42	34.14	10.3912	0.0143	107.8225	0.2147	0.9362	1″
2′	13.62	29.71	50.71	34.05	11.0122	0.0183	112.4572	0.2241	0.899	2″
n1	13.70	29.56	57.97	34.14	8.8443	0.0133	104.3467	0.2077	0.8875	3″
n3	13.54	23.47	55.15	34.40	9.9347	0.0232	123.3294	0.2468	0.8084	4″
3′	13.65	24.85	68.68	34.52	5.7761	0.0128	104.392	0.2082	0.7696	5″

### Appendix A.5. Step 5: Searching of New Food by Unemployed Bees

In this phase, the unemployed bees are selected from outcome of employed bees based on roulette wheel selection. The probability and cumulative probability of each bee provided in Table A11 are determined using Equations (A9) and (A10) and presented in Table A11. The details of unemployed bees are provided in Table A12.

(A9) $$Pr_{i}=\frac{OV_{i}}{\sum_{i=1}^{nb}OV_{i}}$$

(A10) $$CPr_{i}=\overset{i}{\underset{j=1}{\sum }}P_{rj}$$

**Table A11:** Selection of unemployed bees (*UeNo*).

OEBNo.	Pr	CP	Rno	SOEBNo.	UBNo.
1″	0.2177	0.2177	0.8143	4″	u1
2″	0.209	0.4267	0.2435	2″	u2
3″	0.2064	0.6331	0.9293	5″	u3
4″	0.188	0.8211	0.35	2″	u4
5″	0.1789	1	0.1966	1″	u5

**Table A12:** Unemployed bees (*UBNo_i_*).

UBNo.	*V*	*EC*	*F*	*DC*	*MRR*	*OC*	*CC*	*CL*
u1	13.54	23.47	55.15	34.40	9.9347	0.0232	123.3294	0.2468
u2	13.62	29.71	50.71	34.05	11.0122	0.0183	112.4572	0.2241
u3	13.65	24.85	68.68	34.52	5.7761	0.0128	104.392	0.2082
u4	13.62	29.71	50.71	34.05	11.0122	0.0183	112.4572	0.2241
u5	13.86	29.59	53.42	34.14	10.3912	0.0143	107.8225	0.2147

Similar to the searching of new food by the employed bees (step d), the procedure is followed by the unemployed bees to find the new solutions. Table A13 represents the random values of each bee between −1 and 1 and the selected onlooker bee number to calculate the new solution. Table A14 shows the new solutions obtained using the unemployed bees. Each objective value of the unemployed bees shown in Table A12 is compared with the objective values of the new food identified by the unemployed bees shown in Table A14 after converting the multiobjective into a single one. A new solution is generated from the lower and upper limit of the parameter when 50% of the single objective value of the new one is better than the old one. It is shown in Table A15. Two newly generated parameters are obtained because more than 50% of the new solutions (nu2, nu3, nu4 and nu5) are better than the old solutions. It is represented in Table A15.

**Table A13:** Random number and selected unemployed bee for new solution (SUeNo.).

UBNo.			*R_ij_*		*RUBNo(k)*
	*V*	*EC*	*F*	*DC*	
u1	−0.4978	0.1705	0.5075	0.0616	u3
u2	0.2321	0.0994	−0.2391	0.5583	u1
u3	−0.0534	0.8344	0.1356	0.868	u2
u4	−0.2967	−0.4283	−0.8483	−0.7402	u1
u5	0.6617	0.5144	−0.8921	0.1376	u4

**Table A14:** New food by unemployed bees (*NueNo.*).

NUBNo.	*nV*	*nEC*	*nF*	*nDC*	*nMRR*	*nOC*	*nCC*	*nCL*
nu1	13.60	23.23	56.22	34.39	9.6633	0.0219	122.3431	0.2448
nu2	13.64	25.29	51.77	33.86	10.9167	0.0193	122.448	0.2448
nu3	13.65	20.81	53.31	34.93	10.7876	0.0287	130.2479	0.2611
nu4	13.60	27.03	54.48	34.31	9.9926	0.0198	114.8823	0.2293
nu5	12.99	29.54	51.01	34.15	10.3917	0.0265	117.4701	0.2343

**Table A15:** Comparison of new values with unemployed bees.

UBNo.		NUBNo.		Cnt	*V*	*EC*	*F*	*DC*	*MRR*	*OC*	*CC*	*CL*
B.No.	OV	B.No.	OV									
u1	0.668	nu1	0.332									
u2	0	nu2	1	1								
u3	0.4261	nu3	0.5739	2								
u4	0	nu4	1	3	12.41	22.63	63.08	55.7441	8.5554	0.1756	94.066	0.1892
u5	0.0006	nu5	0.9994	4	11.80	20.84	54.58	63.1401	11.4591	0.2395	101.3516	0.2047

### Appendix A.6. Step 6: Replacement of Initial Solution of Bees

The outcome of the employed bees (Table A10) and the outcome of the unemployed bees (Table A16) are combined together and completely replaced with the initialization solution (Table A1 and Table A2). Table A17 represents the replaced solution details. The best parameters and their objective values are stored in the file corresponding to the maximum *OV* value.

**Table A16:** Outcome of unemployed bee solutions.

OUBNo.	*V*	*EC*	*F*	*DC*	*MRR*	*OC*	*CC*	*CL*
1‴	13.54	23.47	55.15	34.40	9.9347	0.0232	123.3294	0.2468
2‴	13.64	25.29	51.77	33.86	10.9167	0.0193	122.448	0.2448
3‴	13.65	20.81	53.31	34.93	10.7876	0.0287	130.2479	0.2611
4‴	12.41	22.63	63.08	55.7441	8.5554	0.1756	94.066	0.1892
5‴	11.80	20.84	54.58	63.1401	11.4591	0.2395	101.3516	0.2047

**Table A17:** Replaced solution.

B.No.	*V*	*EC*	*F*	*DC*	*MRR*	*OC*	*CC*	*CL*	*OV*
1″	13.86	29.59	53.42	34.14	10.3912	0.0143	107.8225	0.2147	0.9995
2″	13.62	29.71	50.71	34.05	11.0122	0.0183	112.4572	0.2241	0.9986
3″	13.70	29.56	57.97	34.14	8.8443	0.0133	104.3467	0.2077	0.9998
4″	13.54	23.47	55.15	34.40	9.9347	0.0232	123.3294	0.2468	0.9954
5″	13.65	24.85	68.68	34.52	5.7761	0.0128	104.392	0.2082	0.9998
1*‴*	13.54	23.47	55.15	34.40	9.9347	0.0232	123.3294	0.2468	0.9954
2*‴*	13.64	25.29	51.77	33.86	10.9167	0.0193	122.448	0.2448	0.9971
3*‴*	13.65	20.81	53.31	34.93	10.7876	0.0287	130.2479	0.2611	0.9907
4*‴*	12.4079	22.6297	63.0816	55.7441	8.5554	0.1756	94.066	0.1892	0.1382
5*‴*	11.8022	20.8382	54.5795	63.1401	11.4591	0.2395	101.3516	0.2047	0.002

### Appendix A.7. Step 7: Stopping Criteria

During this phase, starting from step c to step g, they are repeated for the given number of iterations or until reaching the specified objective value or no further change in the consecutive specified number of iterations. The implementation of the ABC algorithm is shown in Figure 6.
